# Fentanyl compared to buprenorphine for atrial fibrillation ablation analgesia and sedation: a retrospective cross-sectional study

**DOI:** 10.1186/s40780-020-00184-6

**Published:** 2021-01-04

**Authors:** Teruhisa Kinoshita, Mitsunori Harada, Norio Takimoto, Daichi Shibata, Takashi Sakakibara, Mamoru Adachi

**Affiliations:** 1 Department of Pharmacy, Kariya Toyota General Hospital, 5-15, Sumiyoshi-cho, Kariya City, Aichi Prefecture 448-8505 Japan; 2 Department of Cardiology, Kariya Toyota General Hospital, 5-15, Sumiyoshi-cho, Kariya City, Aichi Prefecture 448-8505 Japan

**Keywords:** Atrial fibrillation, Buprenorphine hydrochloride, Catheter ablation, Fentanyl citrate

## Abstract

**Background:**

The effects of general anesthesia with deep sedation and conscious sedation have been compared for sedation management in the perioperative period for radiofrequency catheter ablation of the heart to treat atrial fibrillation. However, there is no consensus as to which drug to use for conscious sedation. This study aimed to investigate analgesic and sedative drugs suitable for perioperative ablation.

**Methods:**

We retrospectively examined 93 patients who underwent atrial fibrillation ablation at Kariya Toyoda General Hospital between December 2017 and April 2019 and investigated differences in the outcomes, such as depth of sedation and postoperative adverse events between the buprenorphine hydrochloride (*n* = 46) and fentanyl citrate (*n* = 47) groups.

**Results:**

The depth of sedation was similar between the two groups, without significant between-group differences in postoperative vomiting. The number of additional injections of thiamylal sodium to manage discomfort and pain during ablation were significantly lower in the fentanyl group. Additionally, the cumulative area product, cumulative total air kerma, 1-year postoperative atrial fibrillation recurrence rate, and postoperative complications were not significantly different between the two groups.

**Conclusions:**

Although there were no significant differences in the efficacy or safety between buprenorphine hydrochloride and fentanyl citrate as analgesics used during atrial fibrillation ablation, intraoperative body movements and patient discomfort could be reduced to a greater extent with the use of fentanyl.

## Background

Ablation for atrial fibrillation (AF) is considered effective for maintaining the sinus rhythm and has been shown to improve quality of life without increasing the risk of complications compared with medical therapy in the wider AF population, with additional mortality and clinical benefits in patients with heart failure.

It has also been suggested that ablation may reduce the risk of ischemic stroke and death [1–3], and therefore, it is widely used. Stable sedation/analgesia for ablation increases patient satisfaction and the success rate of the procedure and is considered essential for preventing complications such as cardiac tamponade and air embolisms. It has been reported that, in AF ablation, there was increased success in pulmonary vein isolation with general anesthesia than with conscious sedation [4, 5]. However, there are facilities where it is difficult for the anesthetist to be present at the time of the ablation; therefore, conscious sedation is often carried out. Furthermore, there is no established consensus regarding which drug to use for perioperative anesthesia management for ablation. Even in the guidelines [6], there are no recommendations for specific drugs.

At the Kariya Toyota General Hospital from December 2017 to July 2018, anesthesia management was carried out using the following drugs: thiamylal sodium, dexmedetomidine hydrochloride, and buprenorphine hydrochloride. However, buprenorphine hydrochloride was switched to fentanyl citrate in August 2018 because of the high frequency of additional use of thiamylal sodium to reduce body movement caused by pain and intraoperative discomfort during myocardial cautery and reduce the high incidence of postoperative vomiting.

In the present study, we compared the efficacy of buprenorphine hydrochloride and fentanyl citrate and investigated analgesic and sedative drugs suitable for perioperative ablation.

## Methods

### Target patients

The study period was 17 months from December 1, 2017, to April 30, 2019. Eligible patients were those who had undergone ablation for AF at our hospital. The group treated with thiamylal sodium, dexmedetomidine hydrochloride, and buprenorphine hydrochloride was the “buprenorphine group,” and the group treated with thiamylal sodium, dexmedetomidine hydrochloride, and fentanyl citrate was the “fentanyl group.”

### Investigation method

From electronic patient charts, we retrospectively analyzed patient characteristics including age, sex, body mass index (BMI), CHADS2 score, CHA2DS2-VASc score, serum creatinine, creatinine clearance (Ccr), left atrial dimension (LAD), left ventricular ejection fraction (LVEF), and cardiac ablation site. Ccr was calculated using the Cockcroft-Gault equation: Ccr (mL/min) = (140-age) × weight / (72 × serum creatinine value) (for female patients, multiplied by 0.85).

Indicators of the effectiveness of ablation included the rate of AF recurrence at 1 year after ablation, postoperative complications, intraoperative cumulative dose area product, and cumulative total air kerma.

Intraoperative sedation was measured using the bispectral index (BIS) [7] and Richmond Agitation-Sedation Scale (RASS), which are defined below.

The BIS is a processed electroencephalographic parameter, which provides a measure of sedation depth on a unitless scale from 0 to 100 (0–40, deep hypnotic state; 40–60, general anesthesia; 60–90, deep-to-light sedation; and 90–100, awake).

The RASS describes the clinical level of sedation (− 5, coma; − 4, deep sedation; − 3, moderate sedation; − 2, mild sedation; − 1, somnolent state; 0, clarity of consciousness; 1, restless; 2, excited; 3, very excited; 4, belligerent), and has been used particularly for assessing the safety of different analgesics.

Side effects were recorded, including the presence of vomiting and the administration of antiemetics. Metoclopramide hydrochloride 10 mg was administered intravenously as a prophylactic at the discretion of the attending physician. Vomiting was included as a side effect if it occurred after the beginning of treatment on the day of ablation.

Patients were followed for 1 year postoperatively to detect recurrence of AF. Patients with post-ablation complications that required prolonged hospital stay or hospitalization within 1 month and required treatment were considered to have complications.

For other sedatives, dexmedetomidine hydrochloride was administered continuously, and thiamylal sodium was temporarily added for the management of body movements and discomfort associated with the procedure. Doses of both drugs were determined at the discretion of the operators.

Sedation for intraoperative electrical defibrillation was performed with thiamylal sodium. To ascertain the number of doses of thiamylal sodium in response to body movements and discomfort caused by the procedure, thiamylal administration at the time of esophageal temperature sensor insertion and before electrical defibrillation was excluded from the count.

Intraoperative sedation was monitored by the cardiologist, and no anesthesiologist was present in the angiography room.

### Statistical methods

For statistical analysis, we used EZR version 3.4.18 (Jichi Medical University Saitama Medical Center, Japan) [8]. Continuous variables are all shown as mean ± S.D. For a comparison of continuous variables, after confirming the normality and distribution of the data, the Mann–Whitney U test and Student’s t-test were used for two-group comparisons, as appropriate. For a comparison of nominal variables, Fisher’s exact test was used. Statistical significance was set at *p* < 0.05.

### Ethical considerations

This study was carried out following the Helsinki Declaration, the “Medical Guidelines for Medical and Health Research Involving Human Subjects,” and the “Guide for the appropriate handling of personal information for medical and nursing care professionals.” Approval was obtained from this hospital’s Ethics Review Committee, and adequate consideration was given for the protection of personal data (Approval No. 517).

## Results

### Patient characteristics

We analyzed the data of 46 patients in the buprenorphine group and of 47 patients in the fentanyl group who underwent AF ablation at our hospital during the study period. The characteristics of the target patients are shown in Table 1.

**Table 1:** Characteristics of the patients who underwent ablation

	Buprenorphine(*n* = 46)	Fentanyl(*n* = 47)	*p* value
Age (years) mean ± SD	64.5 ± 10.2	63.6 ± 9.1	0.66
Sex (M/F)	35/11	32/15	0.49
Body mass index (kg/m^2^) mean ± SD	24.8 ± 4.2	23.9 ± 5.4	0.67
CHADS_2_ score median (min-max)	1 (0–4)	1 (0–5)	0.99
CHA_2_DS_2_-VASc score median (min-max)	2 (0–5)	2 (0–7)	0.87
Creatinine clearance (mL/min) mean ± SD	86.3 ± 27.1	81.2 ± 26.7	0.36
Left atrial dimension (LAD)(cm) mean ± SD	3.71 ± 0.57	3.86 ± 0.65	0.27
Left ventricular ejection fraction (LVEF) (%) mean ± SD	69.24 ± 6.15	63.89 ± 9.19	< 0.01
Average Drug Use (mg) mean ± SD	0.18 ± 0.05	1.06 ± 0.35	–
Dexmedetomidine hydrochloride (μg) median (min-max)	107.2 (46.0–201.6)	117.2 (61.6–282.4)	0.14
Patients who received cardioversion No. of patients (percentage of patients)	25 (54.3%)	26 (55.3%)	1.00
**Type of AF**			
Paroxysmal. No. of patients (percentage of patients)	31 (67.4%)	29 (61.7%)	0.83
Persistent No. of patients (percentage of patients)	13 (28.2%)	15 (31.9%)	
Long lasting persistent No. of patients (percentage of patients)	2 (4.3%)	3 (6.4%)	
**Ablation method**			
PVI No. of patients (percentage of patients)	31 (67.4%)	31 (66.0%)	0.45
PVI + CTI ablation No. of patients (percentage of patients)	9 (19.6%)	13 (27.7%)	
Other No. of patients (percentage of patients)	6 (13.0%)	3 (6.4%)	

*No.* number, *PVI* Pulmonary vein isolation, *CTI* cavo-tricuspid isthmus line

Although LVEF was significantly higher in the buprenorphine group, it was with normal parameters in both groups, and there were no significant differences in terms of other parameters. The patients were relatively young (the average patient age was 64 years old), and their renal function was normal. Most patients had a relatively low risk of stroke caused by AF.

There was no difference in the intraoperative use of dexmedetomidine hydrochloride between the two groups.

### Treatment efficacy

A comparison of treatment effects is summarized in Table 2**.** The sedation index is summarized in Table 3, and adverse events are summarized in Table 4. There was no significant difference in the rate of recurrent AF between the buprenorphine and fentanyl groups 1 year after surgery.

**Table 2:** Treatment outcomes and exposure doses

	Buprenorphine (*n* = 46)	Fentanyl (*n* = 47)	*p* value
Recurrence of AF No. of patients (percentage of patients)	10 (21.7%)	14 (29.8%)	0.48
Complications No. of patients (percentage of patients)	0 (0%)	1 (2.1%)^a^	1.00
Cumulative DAP (fluoroscopy) (mGycm^2^) median (min-max)	22,298.5 (9749–188,652)	25,100.0 (8750–95,081)	0.20
Cumulative DAP (exposure) (mGycm2) median (min-max)	3779 (109–22,622)	3260 (166–11,013)	0.38
Cumulative total air kerma (mGy) median (min-max)	286.4 (95.3–2192.4)	268.5 (84.9–1105.0)	0.51

^a^ Pseudoaneurysm

*AF* atrial fibrillation, *DAP* dose area product

**Table 3:** Sedation index during ablation

	Buprenorphine(*n* = 46)	Fentanyl(*n* = 47)	*p* value
Average BIS median (min-max)	82.8 (59.0–95.5)	76.9 (62.5–95.6)	0.02
Average RASS score median (min-max)	−1.38 (−4.00–0.00)	−2.00 (− 4.00–0.00)	0.11
No. of additional administrations of thiamylal median (min-max)	1 (0–6)	1 (0–4)	< 0.01
Thiamylal Sodium usage (mg) median (min-max)	75 (0–450)	25 (0–250)	< 0.01

*BIS* bispectral index, *No.* number, *RASS* Richmond Agitation-Sedation Scale

**Table 4:** Adverse events and antiemetic administration after ablation

	Buprenorphine (*n* = 46)	Fentanyl (*n* = 47)	*p* value
No. of patients with vomiting No. of patients (percentage of patients)	14 (30.4%)	7 (14.9%)	0.09
No. of patients receiving prophylactic metoclopramide hydrochloride No. of patients (percentage of patients)	31 (67.4%)	24 (51.1%)	0.14

*No.* number

There was also no significant difference in postoperative complications, the exposure to the cumulative dose area product, and cumulative total air kerma between the two groups.

As an indicator of safety with different analgesics, intraoperative BIS values were 82.8 (59.0–95.5) in the buprenorphine group and significantly lower, 76.9 (62.5–95.6), in the fentanyl group (*p* = 0.02), but the RASS score was not significantly different between the two groups.

The median number of additional doses of thiamylal sodium, an indicator of intraoperative pain and discomfort, was similar to that of the fentanyl group (once, [0–4]) and the buprenorphine group (once [0–6]; p = < 0.01), but the distribution was less in the fentanyl group, and the difference was significant. There was no significant difference in adverse events (postoperative vomiting) between the two groups. There was also no significant difference in metoclopramide hydrochloride administration.

## Discussion

In the present study, we examined whether different anesthesia management agents used for ablation affect outcomes in terms of efficacy (the rate of recurrence and complications of AF (atrioventricular block, cardiac tamponade, fistula in left esophagus, and others)) and safety (depth of sedation, number of additional doses of thiamylal sodium administered, and frequency of vomiting). The number of additional doses of thiamylal sodium, an indicator of intraoperative pain and discomfort, administered were significantly lower in the fentanyl group than in the buprenorphine group. The average usage of buprenorphine hydrochloride in the buprenorphine group was 0.18 mg. In contrast, the average usage of fentanyl citrate in the fentanyl group was 1.06 mg. In the Pharmaceutical Guidelines of the Japanese Society of Anesthesiologists [9], the respective analgesic effects of buprenorphine hydrochloride and fentanyl citrate are approximately 33–40 times and 50–100 times that of morphine.

Fentanyl citrate generally has a titer twice of that of buprenorphine hydrochloride, indicating that fentanyl citrate is used in higher quantities. The BIS was significantly lower in the fentanyl group, but the difference was small (approximately 3.8) and could be considered of minor clinical significance. Both groups were in the category of deep to mild sedation. There was no difference in the RASS scores between the two groups, and the sedative effect was considered to be nearly equivalent. The use of fentanyl citrate as an analgesic in sufficient quantities is expected to reduce intraoperative discomfort, pain, and body movement without any deeper sedation than necessary, representing a more reliable and safe option for the physician. However, with respect to postoperative vomiting, there was no significant difference between the two groups of patients who received metoclopramide as an antiemetic agent. In a meta-analysis of opioid adverse events [10], vomiting was more common with buprenorphine hydrochloride than with fentanyl citrate.

Buprenorphine hydrochloride is a long-acting drug (with a half-life of approximately 10 h). Conversely, fentanyl citrate is shorter acting (with a half-life of 30 min to 1 h) [9]. Hence, we switched from buprenorphine hydrochloride to fentanyl citrate considering that the effects of fentanyl citrate would dissipate while the effects of dexmedetomidine hydrochloride would remain, thereby reducing postoperative vomiting. In a previous study using fentanyl as an analgesic [11], approximately 13% of patients were found to have vomiting, and the frequency of vomiting in the fentanyl group was as expected. There was no report of buprenorphine use during ablation, but the frequency of vomiting that occurred from other uses varied widely, ranging from a few percent to approximately 50%, as reported [12–15]. In this study, there was a difference in vomiting (approximately 30% in the buprenorphine group and approximately 15% in the fentanyl group), but the difference was not statistically significant. One possible explanation for the lack of a significant difference between the two groups was the higher use of fentanyl compared to that of buprenorphine.

The most important outcomes of AF ablation to consider are the rate of AF recurrence and complications of AF. A previous study [16] reported a recurrence rate of 30–50% at 1 year postoperatively for AF. Although the current study was conducted with a small group, the recurrence rate was not significantly different from the previous study. There were few complications in this study, and they were well managed. Therefore, the combination of fentanyl citrate, thiamylal sodium, and dexmedetomidine hydrochloride may be a useful option in the management of anesthesia during AF ablation, particularly because of the reduction of intraoperative body movements with this approach.

This study has potential limitations. All patients in the present study underwent radiofrequency ablation; therefore, it is unclear whether our results can be extrapolated to the management of anesthesia during cryoablation or hot balloon ablation procedures. Additionally, the use of different catheter and respiratory management devices from one facility to the next may result in varied outcomes. As this study was carried out retrospectively at a single center with a small sample size, future randomized comparative trials on a larger scale would be necessary.

## Conclusions

In conclusion, both fentanyl citrate and buprenorphine hydrochloride are useful in the management of anesthesia during AF ablation, suggesting that the use of fentanyl citrate may decrease intraoperative patient motion and discomfort.

## Data Availability

The datasets analyzed during the current study are available from the corresponding author on reasonable request.

## Abbreviations

AF: Atrial fibrillation
BIS: Bispectral index
BMI: Body mass index
Ccr: Creatinine clearance
CTI: Cavo-tricuspid isthmus line
DAP: Dose area product
LAD: Left atrial dimension
LVEF: Left ventricular ejection fraction
PVI: Pulmonary vein isolation
RASS: Richmond Agitation-Sedation Scale
